# A new model for COVID-19 control and its implementation in the city of Honghu, China: a case report

**DOI:** 10.1186/s13756-021-00899-7

**Published:** 2021-02-25

**Authors:** He-ran Wang, Meng-chun Gong, Jing-Yuan Sun, Jian Sun, Yi Guo, Li Liu, Hong Zhu

**Affiliations:** 1 Department of Medical Quality Management, Nanfang Hospital, Southern Medical University, Guangzhou, 510515 China; 2 Department of Infectious Diseases, Nanfang Hospital, Southern Medical University, Guangzhou, 510515 China; 3 Health Management Institute, Nanfang Hospital, Southern Medical University, Guangzhou, 510515 China

**Keywords:** COVID-19, Prevention and management, Honghu model, Pandemic

## Abstract

**Background:**

Novel coronavirus pneumonia has been the most serious worldwide public health emergency since being identified in December 2019. The rapid spread of the pandemic and the strong human to human infection rate of COVID-19 poses a great prevention challenge. There has been an explosion in the number of confirmed cases in several cities near Wuhan, including the highest in Honghu, Jinzhou. Owing to the limited admission capacity and medical resources, increasing numbers of suspected cases of COVID-19 infection were difficult to confirm or treat.

**Case presentation:**

Following the arrival of the Guangdong medical aid team on 11 February, 2020, COVID-19 care in Honghu saw changes after a series of solutions were implemented based on the ‘Four-Early’ and ‘Four-centralization’ management measures. The ‘Four-Early’ measures are: early detection, early reporting, early quarantine, and early treatment for meeting an urgent need like the COVID-19 pandemic. ‘Four-centralization’ refers to the way in which recruited medical teams can make full use of medical resources to give patients the best treatment. These solutions successfully increased the recovery rate and reduced mortality among patients with COVID-19 in Honghu.

**Conclusions:**

This management strategy is called the ‘Honghu Model’ which can be generalized to enable the prevention and management of COVID-19 worldwide.

## Background

### Why a new management model was needed in Honghu

An outbreak of viral-pneumonia caused by Coronavirus Diseases 2019 (COVID-19) occurred in Wuhan in early December 2019 and rapidly spread to the whole country [1]. Honghu is a county-level city with a population of 900,000, an underdeveloped economy and limited medical resources. Honghu is geographically near Wuhan, the epicentre of the pandemic. There is significant mobility between the cities of Honghu and Wuhan. From 16 to 23 January 2020, approximately 49,000 individuals travelled to Honghu from Wuhan, which resulted in a large number of potentially imported COVID-19 cases in Honghu. These high-risk passengers from Wuhan could not be quickly traced, and evaluated for symptoms of COVID-19 infections.

## Case presentation

Before the medical team was deployed (12 February, 2020), 262 cases of COVID-19 were diagnosed in Honghu, among which many were in a severe condition, with the case fatality rate in Honghu being higher (1.94% [Honghu] vs. 1.23% [outside Wuhan]), while the recovery rate being lower (0% [Honghu] vs. 14.90% [outside Wuhan]). Prior to the medical team’s arrival, all the COVID-19 cases in Honghu were under treatment and none of them had fully recovered. Moreover, because of a severe lack of nucleic acid testing platforms in Honghu, large numbers of potentially infected patients were difficult to identify.

Therefore, the COVID-19 nucleic acid test samples of suspected cases had to be sent to Wuhan or Jingzhou as soon as possible, which took a minimum of three hours physical transit before testing. Moreover, only two hospitals in Honghu were equipped with CT scanning equipment. The lack of CT scanning equipment greatly delayed the confirmation of suspected cases.

Patients diagnosed with COVID-19 were admitted and spread out among seven medical institutions and 21 township health centres in Honghu during the early COVID-19 outbreak. Of these, only Honghu People’s Hospital was capable of treating patients with infectious diseases, and the patients at other hospitals could not receive standardized treatment for infectious diseases due to a lack of experience. Therefore, there was a higher proportion of severely-ill patients in Honghu than the average for the rest of Hubei. Compared with other counties in Jingzhou city, Honghu had the most serious pandemic area, accounting for approximately 25% of patients with severe symptoms in Jinzhou city.

Since the Guangdong medical aid team was deployed, with the support of the government, a series of administrative measures were performed forming a new model called the ‘Honghu Model’ that effectively contained the transmission of COVID-19 and increased the recovery rate from infections in Honghu.

## How was the new model built and how did it perform in Honghu?

Since February 12th, 2020, given the information for the local pandemic, the Guangdong medical aid team initiated a unique management system for pandemic control. A series of measures underlying the system were taken to successfully control the pandemic and increase the recovery rate of infection cases.

### A ‘Four-Early’ measures for timely prevention and control

The ‘Four-Early’ measures is extensively acknowledged by most medical personnel involved in pandemic control in China [2]. The ‘Four-Early’ measures is the core of the Honghu model, which contains four aspects, namely, early detection, early reporting, early quarantine, and early treatment.

To achieve the requirements of the ‘Four-Early’ measures, first, a personal electronic health reporting system was launched with technical assistance from the Digital China Health Technologies Company (China). The system was available to all residents of Honghu who accessed it to fill in personal health information and pandemic history (in particular whether they had contact with people from Wuhan or with confirmed COVID-19 patients) and uploaded the information to the system via their mobile devices. Furthermore, by analysing the information uploaded from the personal electronic health reporting system, the government could communicate with individuals in the community when they were predicted to be at high risk of being infected. Community workers were assigned to visit residents at high risk and instruct them to self-isolate. In addition, these residents were requested to report body temperature, where they had been within fourteen days and whether they had contact with confirmed COVID-19 patients. The feedback system between residents and the government played an important role in isolating suspected people with a high risk of infection as soon as possible. Thus, the government could easily discover and monitor the trends of the COVID-19 pandemic in Honghu. Second, with the cooperation of the Jinyu Medical Laboratory Technology Company and the laboratory department of the Honghu People’s Hospital, a nucleic acid detection platform was set up within two days. This meant that suspected cases could be tested timely, rather than shipping samples to Wuhan for testing. At the same time, computed tomography (CT) equipment donated by the Guangzhou Tencent Fund (China) was installed within 36 h. Subsequently, nucleic acid testing and further CT detection for people who need to be tested were soon utilized. This platform greatly increased the speed of daily nucleic acid screenings in Honghu.

### A ‘Four-Centralization’ requirement for the treatment of infectious patients

The four aspects of ‘Four-Centralization’ are ‘patients,’ ‘medical staff,’ ‘medical resources’ and ‘therapeutic schemes’ working as a coalition during outbreaks of infectious diseases. Each element played a unique role in the system. I, Patients were consolidated centrally and admitted to the designated hospital. II, Local medical staff set up medical teams specific to COVID-19. III, Existing medical resources such as ventilators or first-aid medical supplies were distributed to the designated hospitals receiving COVID-19 infected patients. IV, All patients with suspected COVID-19 were admitted and given the best appropriate treatment. As required by the ‘Four-Centralization,’ the medical aid team carried out a series of measures.

**Fig. 1:**
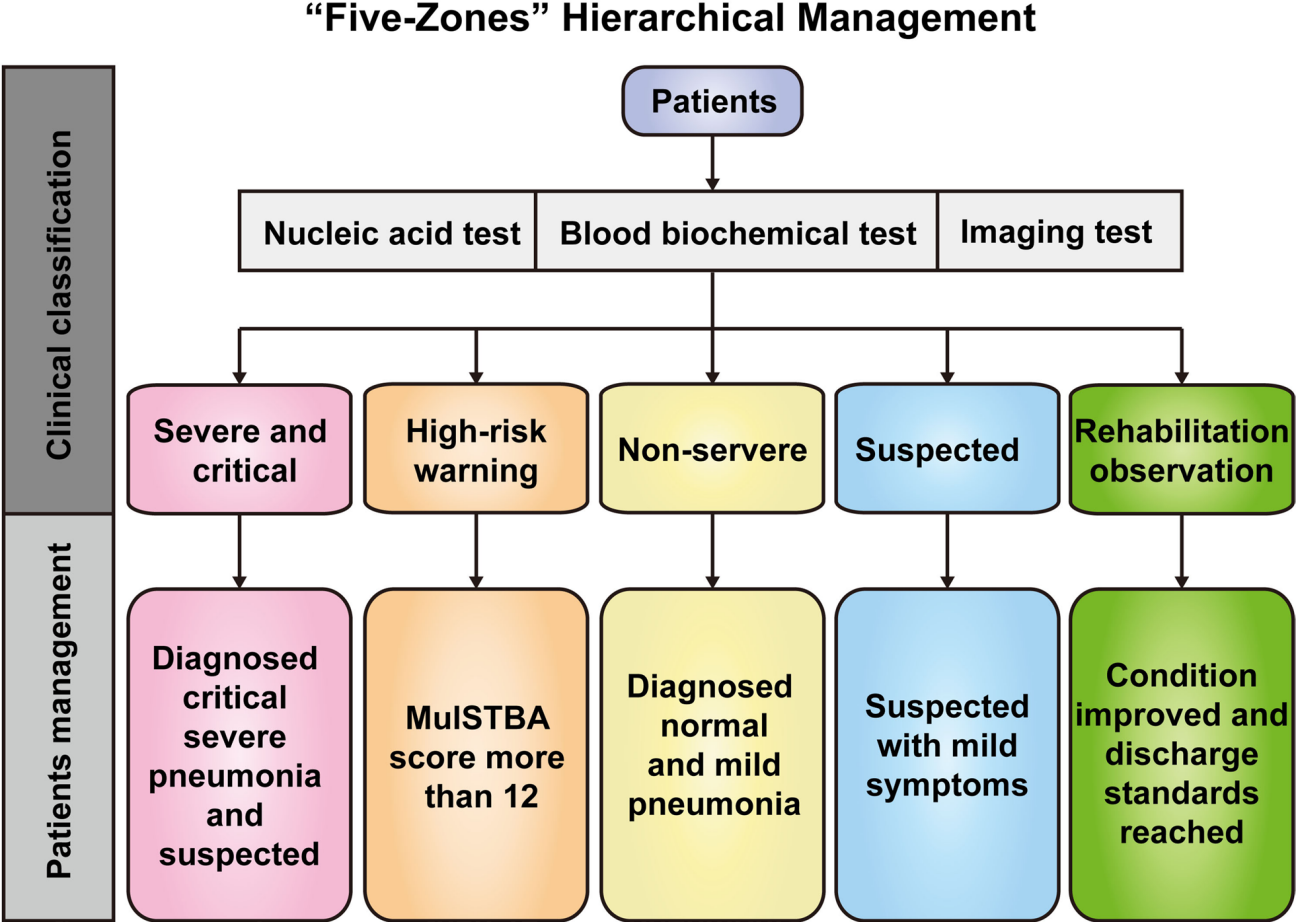
The schematic of a ‘Five-zones’ hierarchical management. The hospitals in Honghu that specialized in receiving COVID-19 patients were re-divided. The ‘red zone’ received only cases with potentially fatal or severe pneumonia. The ‘yellow zone’ mainly received cases with common or mild pneumonia, while the ‘blue zone’ received suspected cases with mild symptoms. Recovered cases would be transferred to the ‘green zone’ for medical observation. The ‘Five-Zone’ management of organization and coordination of medical wards realized targeted management and treatment for patients with different levels of disease severity

First, patients with COVID-19 were consolidated centrally to nine designated hospitals in Honghu. The nine hospitals were divided into five zones—red, orange, yellow, blue, and green—to receive patients exclusively according to different illness statuses (Fig. 1). The ‘red zone’ received only cases with potentially fatal or severe pneumonia, while the ‘orange zone’ received cases with a high risk mulSTBA score of more than 12, which was calculated on the basis of a multivariate logistic regression model in order to predict mortality, with a weighted score that included multilobular infiltrates [3, 4], the aged, and those with confirmed comorbidities. The ‘yellow zone’ mainly received cases with common or mild pneumonia, while the ‘blue zone’ received suspected cases with mild symptoms. Recovered cases would be transferred to the ‘green zone’ for medical observation. The ‘Five-Zone’ management of organization and coordination of medical wards realized targeted management and treatment for patients with different severities of illness. After these measures were put in place, the recovery rate gradually increased to 31.1% from no cured cases before the medical team arrived.

Second, the clinical information management and the analysis system covering 9 designated hospitals was established. Through the system, the experts could acquire all needed clinical data about patients at any time for consultations. Additionally, the analysis of the clinical data in the system provided guidance for decision-making on treatment and formed the basis for prevention and control policy and allocation of medical resources.

Third, for severely ill patients or patients with a high risk of death, the Guangdong medical aid team prepared an expert consultation centre to establish a remote consultation system that covered all designated hospitals in the city. Through this consultation, an average of 10 to 20 video consultations per day could be provided. In addition, daily quantitative assessments of the death risk were performed according to the Clinical Features Predicting Mortality Risk in Patients with Viral Pneumonia [4]. Then an expert consultation would be organized for patients with severe conditions immediately depending on the assessments. Since the daily assessments and expert consultations began, the mortality rate was reduced from 3.05% (8/262) to 2.63% (10/380).

The Guangdong medical aid team rearranged the ICU at the People`s Hospital in Honghu to increase the number of beds by 20 within four days for severe patients. Building the Honghu ‘Xiaotangshan’ hospital with the assistance of the Honghu government within ten days and implementing the ‘five-zone’ system increased the number hospital beds from 332 to 951. After these measures were put in place, the recovery rate dramatically increased up to 31.1% from when the medical team arrived.

### Long-term follow up and full-chain care for recovered patients

After COVID-19 patients had recovered and were discharged, they were required to be quarantined at home for 14 days for medical observation and health management. For those who did not have the appropriate environment for home isolation, it was conducted at a local centralized isolation point. In addition, the recovered cases were educated to keep their room well-ventilated, pay attention to hand hygiene, and take temperature measurements in the morning and evening. If the recovered patients had clinical manifestations such as fever and cough, they were transferred to designated hospitals for further treatment. After the isolation period, further examination of health conditions was carried out to prevent recurrence, and only thereafter the isolation period could be terminated.

### Well-trained medical personnel

Upon their arrival in Honghu, the Guangdong medical aid team launched training protocols for all medical personnel. The training entailed the diagnosis and treatment of COVID-19 infections, self-protection and use of traditional Chinese medicine. Firstly, each of the medical personnel were assigned to their own isolation points in Honghu. Secondly, the Guangdong medical aid team arranged a video conference to train medical personnel in Honghu, both theoretically and practically, with particularly emphasise on the importance of standardized precautions [5]. Thirdly, experienced infection control experts conducted and supervised pre-engagement training to ensure that all personnel followed the correct procedures. The operation process followed by the medical personnel was supervised by experts. No medical personnel were found to be infected during, or after, training.

### What did we learn from this new model?

**Fig. 2:**
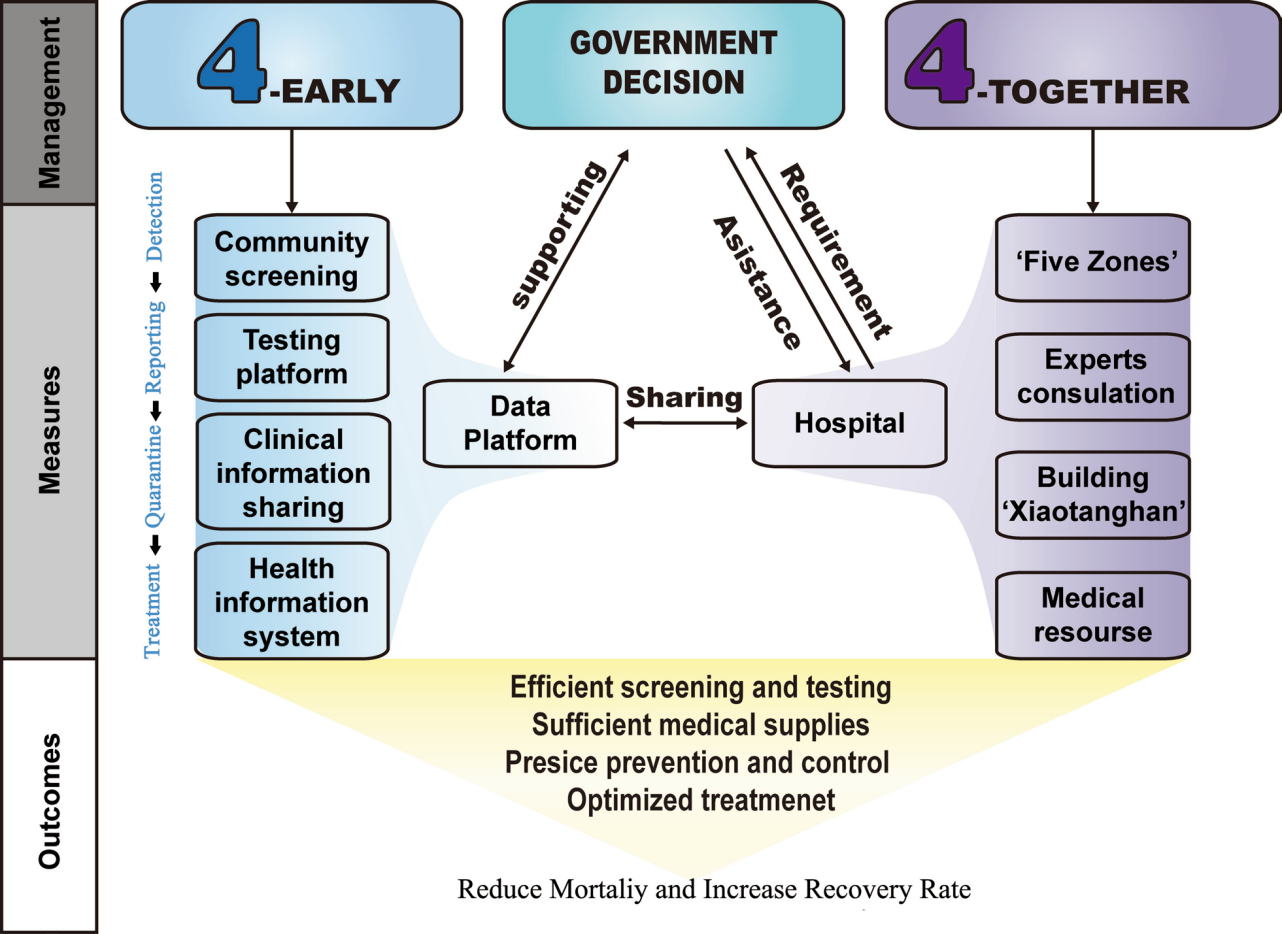
The schematic of ‘Honghu Model’. Under the guidance of the ‘Four-Early’ and ‘Four-Centralization’ measures, a series of solutions were taken to control the pandemic COVID-19. The information sharing system bridged government and individuals, and government and hospital. The health and clinical information platform helped the government to carry out effective measures for patient isolation with a high risk of infection and helped experts to conduct consultation on patients with a high risk of death, respectively. The measures taken contributed to the sufficient medical supplies, rapid testing and precise prevention and control

The management strategy of the ‘Honghu Model’ for the COVID-19 pandemic practically demonstrates the core of the ‘Four-Early’ and ‘Four-Centralization’ measures. The ‘Honghu Model’ systematically integrates the four core elements, the government, experts, information, and coordination in pandemic control. Every core element plays a unique role in infection control and treatment of infected patients (Fig. 2).

The government should take responsibility for general infection control, including information collection, distribution of resources, training, education, and decision-making with regard to the infection control policy at national level [6]. Effective prevention and control of infection requires data sharing via a suitable platform [7]. Under such special circumstances, this big data sharing system is known as the ‘eyes’ of a city. It is essential to launch a comprehensive data platform that links the government decisions, community residents' health and medical resources. Big data has a certain role in the early warning of infectious diseases as it provides the references for scientific administrative prevention and control, decision-making on precise implementation and prediction of high risk of infection. The government should also establish a close connection with the hospitals, communities, and the enterprises engaged with medical resource production. By collecting data from different platforms, such as the health information system or community reports and pandemic data from hospitals, the government needs to respond quickly to find solutions, such as supervision of those with high risk of infection, reasonable allocation of medical resources according to the situation of the pandemic, and publicizing the importance of pandemic self-protection.

In terms of the severity of symptoms in confirmed COVID-19 patients, strengthening the cooperation between local hospitals such as sharing clinical data and providing expert remote consultation at hospital level, is essential. Cooperation between hospitals in the ‘Honghu model’ had been proven to be effective in reducing mortality rates. First, the information about the updated number of infections in the hospital as a guide for medical resources allocation so that every patient can receive adequate medical supplies. Second, expert remote consultation can help local doctors diagnose and share their clinical experience of COVID-19 infection ensuring each patient has the most suitable individualized treatment. The medical personnel can receive distance medical education and training through the clinical data sharing platform so that all procedures for medical care of COVID-19 infection can be standardised. This cooperation realises clinical multi-disciplines communication that improved the understanding of COVID-19.

The ‘Honghu model’ proved that the ‘Five-Zones’ division of patients depending on their disease severity, improves the prevention and control of infection at patient level. It means that triage can optimize the treatment of patients with COVID-19 infections, which also reduces the probability of cross propagation. In particular, critically or severely affected patients require more medical care such as daily assessments and expert consultation. Additionally, it is necessary for recovered patients to accept isolation at a designated place or at home for 14 days. Successful prevention and control of the pandemic requires the participation of every individual. Everyone should take responsibility for themselves and contribute to prevention and control of the COVID-19 pandemic.

## Conclusion

Even though the COVID-19 pandemic seriously threatens the lives and health of every person, it can be prevented as long as people take effective prevention and control measures. The ‘Honghu Model’ strategy initiated by Guangdong’s medical aid team proved to be effective for the prevention and control of COVID-19 infection. We hope the management strategy of the ‘Honghu Model’ provides a reference for other county-level cities with outbreaks and helps the prevention and control of the COVID-19 pandemic.

## Data Availability

All data in the manuscript can be made available upon request.

## Abbreviations

COVID-19: Coronavirus Diseases 2019
CT: Computed tomography
